# Single Copy Oligonucleotide Fluorescence In Situ Hybridization Probe Design Platforms: Development, Application and Evaluation

**DOI:** 10.3390/ijms22137124

**Published:** 2021-07-01

**Authors:** Guanqing Liu, Tao Zhang

**Affiliations:** 1 Jiangsu Key Laboratory of Crop Genomics and Molecular Breeding, Key Laboratory of Plant Functional Genomics of the Ministry of Education, Agricultural College of Yangzhou University, Yangzhou 225009, China; liuguanqing@zhangtaolab.org; 2 Jiangsu Key Laboratory of Crop Genetics and Physiology, Jiangsu Co-Innovation Center for Modern Production Technology of Grain Crops, Yangzhou University, Yangzhou 225009, China; 3 Institutes of Agricultural Science and Technology Development, Joint International Research Laboratory of Agriculture and Agri-Product Safety of the Ministry of Education, Yangzhou University, Yangzhou 225009, China

**Keywords:** oligo-FISH, probe design, cytogenetics, genome research

## Abstract

Oligonucleotides fluorescence in situ hybridization (Oligo-FISH) is an emerging technology and is an important tool in research areas such as detection of chromosome variation, identification of allopolyploid, and deciphering of three-dimensional (3D) genome structures. Based on the demand for highly efficient oligo probes for oligo-FISH experiments, increasing numbers of tools have been developed for probe design in recent years. Obsolete oligonucleotide design tools have been adapted for oligo-FISH probe design because of their similar considerations. With the development of DNA sequencing and large-scale synthesis, novel tools have been designed to increase the specificity of designed oligo probes and enable genome-scale oligo probe design, which has greatly improved the application of single copy oligo-FISH. Despite this, few studies have introduced the development of the oligo-FISH probe design tools and their application in FISH experiments systematically. Besides, a comprehensive comparison and evaluation is lacking for the available tools. In this review, we provide an overview of the oligo-FISH probe design process, summarize the development and application of the available tools, evaluate several state-of-art tools, and eventually provide guidance for single copy oligo-FISH probe design.

## 1. Introduction

Fluorescent in situ hybridization (FISH) is a powerful technique to detect and locate a specific DNA sequence on a chromosome. Since the emergence of in situ hybridization in 1969 and the subsequent development of FISH [1,2,3], this technique has continued to be used, modernized, and applied to various cytogenetic researches [4,5]. In the past decade, many improvements have been made to enhance the FISH assay. In addition, with the development of DNA sequencing and synthesis technologies, a new generation of FISH techniques, called oligonucleotide fluorescence in situ hybridization (oligo-FISH), which utilizes single-copy oligonucleotides (oligos) as FISH probes, has been established. In recent years, increasing numbers of researchers have embedded this method in their studies [6,7,8].

Oligo-FISH probes offer many advantages compared with conventional probes derived from ribosomal DNA (rDNA), bacterial artificial chromosome (BAC) sequences, or repetitive sequences. Although rDNA and many other tandemly repeated sequences can generate strong FISH signals, they are not suitable for comparative cytogenetic studies because of their prevalence and diversity among different species [9,10,11]. Probes designed from large-insert genomic clones such as BACs generate strong background signals because of the mass of repetitive sequences in the genome, especially in those large complex plant genomes [12,13]. In contrast, synthetic oligo probes derived from a single copy region of genome allow for hybridization to precisely defined targets without the interference of repetitive sequences, and are easy to maintain.

Recently, many advances have been made regarding FISH assays; however, little progress has been made in developing computational tools that support the design of FISH probes, especially for oligo-FISH. Most of these tools are only used to design oligo probes for bacterial rDNA or microarrays, which might be not suitable for oligo-FISH. Although repetitive sequences-based oligo-FISH methods like nondenaturing FISH (ND-FISH) show the advantage in identifying chromosomes or specific segments in genome [14,15,16,17]. FISH based on single copy oligo probes enables chromosome-specific painting and more application scenarios [18,19,20]. To design oligo probes that fulfill the requirements of single copy oligo-FISH, one of the most important processes is to exclude repetitive sequences present in the target genome [8,21,22,23]. Other considerations, such as probe length and thermodynamic properties, are also important [24,25,26]. Here, we introduced the key aspects that should be considered during the probe design process. We also summarize the development and applications of the computational tools that are used for single copy oligo-FISH probe design. In addition, we evaluated several common tools and provide comprehensive suggestions for researchers who need to develop oligo probes for their experiments. We anticipate that our review will provide a reference for wet-lab scientists and suggestions for the development of single copy oligo-FISH and corresponding probe design tools.

## 2. Key Aspects of Oligo-FISH Probe Design

**Figure 1:**
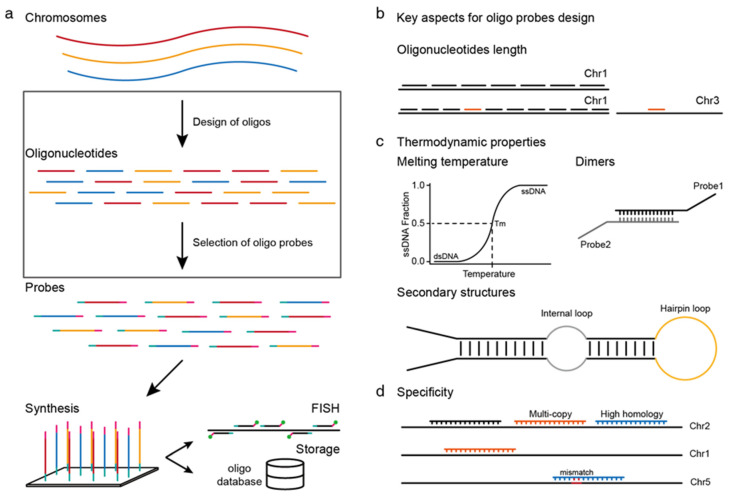
Workflow and the key aspects of oligonucleotide fluorescence in situ hybridization (oligo-FISH) probe design. (**a**) Flow chart of oligo-FISH probe design. Oligos are first designed from target genome, then oligo probes are selected and synthesized for FISH experiments or storing as permanent resources. (**b**–**d**) Key aspects of the design of oligonucleotide probes. (**b**) Oligonucleotide length. (**c**) Thermodynamic properties include melting temperature, formation of dimers and secondary structures. (**d**) Specificity.

A successful oligo-FISH experiment requires the synthesis of massive amounts of single-copy oligos as hybridization probes from the genome (Figure 1a). The pivotal process of oligo-FISH is the design of the single-copy oligo probes. The robustness of an oligo probe is influenced by multiple aspects. To better understand the principle of oligo-FISH probe design, here, we summarized several key aspects that determine the effect of oligo probes on FISH experiments.

### 2.1. Oligonucleotide Length

The stringency, hybridization stability, and efficiency of an oligonucleotide are directly associated with its length [24]. In theory, longer oligonucleotides are usually more unique in the target genome (Figure 1b), because an extra nucleotide increases the uniqueness of an oligonucleotide by a factor of four and enhance the mismatch tolerance [27]. However, longer oligo probes may increase the possibility of forming hairpins, thus reducing the binding efficiency [24]. Besides, the density of a probe dataset decreases when designing longer oligo probes in specific regions or on the genome scale (Figure 1b). For instance, the number of oligos in a given region with shorter length is more than the number of oligos with a longer (45nt) length in the same region; consequently, shorter oligo probes produce increased FISH signals [28].

### 2.2. Thermodynamic Properties

Melting temperature (Tm) is an important factor that affects the hybridization of a DNA probe. The Tm is the temperature at which half of the oligonucleotides are paired with their complementary sequence to forming a duplex (Figure 1c) [26]. Inaccurate estimations of the Tm of oligos might result in unwanted binding results. Thus, to estimate the Tm of a probe exactly, several factors are needed to be assessed, such as the GC content, sodium concentrations, and nearest neighbor (NN) interactions [25,29,30]. In early studies, researchers built the Tm estimation models based on the GC content [31,32]. However, such models are not accurate enough. In 1979, salt molarity and the formamide concentration were incorporated to calculate the Tm [33]. Later, with the addition of NN parameters and oligonucleotide features, prediction models of Tm became more accurate [26,34,35,36,37,38]. In general, Tm estimation based on NN parameters shows better performance than other features [25,26,39].

Other important factors that need to be considered when designing oligo probes are the formation of secondary structures and dimers. If single-stranded oligonucleotides fold and form secondary structures, such as hairpin loops, they may fail to hybridize to the target sites (Figure 1c) [40,41]. Several key features are required for DNA secondary structure predictions, such as salt concentrations, loops, dangling ends, and internal/terminal mismatches [26,42,43,44]. When folding, DNA often forms different loops, which have a marked impact on the hybridization of oligonucleotides to their intended target sites. Therefore, the identification of loop motifs, which produce hairpin loops, internal loops, or bulge loops, are important for secondary structures prediction [39]. Mismatches that lie at both internal and terminal positions of oligonucleotides affect the stability of a duplex, which should be checked up when predicting secondary structures [43]. Just like the formation of secondary structures, one oligo probe might also hybridize with another one to form a dimer, which limits the binding between the oligos and their target DNA sequences (Figure 1c) [45]. The combination of salt molarity, oligonucleotide features and NN parameters will help to predict dimer formation. In general, secondary structures and dimers should be carefully checked during oligo probe design.

### 2.3. Specificity

The most important aspect that determines the success of a FISH experiment based on oligos is the specificity of the probes. Oligo probes should only bind to their intended target DNA. Oligo probes with multiple target sites in genome lead to high background signals, and eventually fail to produce the correct signals in a FISH experiment (Figure 1d). To design efficient single-copy oligo probes, one important aspect is to select a well-assembled reference genome, which covers a major part of the repetitive sequences. In addition, complete annotation of repeat elements in the genome is also vital to help eliminate potential multi-copy oligos. At present, the most commonly used strategy for single-copy oligo detection is dependent on sequence alignment. Common alignment tools such as BLAST [46] or next-generation sequencing (NGS) aligners, such as Bowtie2 [47] and BWA [48], are useful to remove repetitive sequences. However, plant species usually contain complex genomes, which comprise various repetitive elements. For those plants with large genomes, such as maize and wheat, potential repetitive sequences might not be well documented. When designing oligo probes in these genomes, de novo repeats detection and filtering based on shotgun sequences is another approach that can be used to improve the specificity of oligos [49,50,51]. Moreover, it should be noted that oligos might bind to unintended sites with minor mismatches (Figure 1d). Therefore, oligos with multiple homologous sequences in the genome should be monitored carefully. Nevertheless, specificity examination using just the sequence identity is insufficient, because both an oligo’s length and thermodynamic properties influence its specificity [45]. Therefore, all the aspects mentioned above should be considered during the oligo probe design process.

## 3. Development and Application of Oligo-FISH Probe Design Tools

With the development of FISH technology, oligonucleotide-based probes have gradually become the new-generation FISH probes in animals, plants and bacteria [4,52,53]. However, higher demand for oligonucleotide probes for FISH experiments has not resulted in the rapid development of oligo probe design tools, especially for the tools and platforms that can design genome-scaled oligo-FISH probes. Under these circumstances, we systematically summarized the development of tools or web platforms for oligo probe design and discussed their applications in various species or types of researches (Table 1).

OligoArray is a popular program that allows the design of oligonucleotides at the genomic scale. It was first developed in 2002 [61] and further updated in 2003 [54]. OligoArray analyzes the thermodynamics of hybridization to predict secondary structures and the Tm, and then calculates the specificity of length-adjusted oligonucleotide probes according to both their sequence similarity and thermodynamic properties. OligoArray was originally implemented for DNA microarrays and was applied later for FISH probe designs. The Wu laboratory and Rouillard laboratory built a platform called Oligopaint (https://oligopaints.hms.harvard.edu/; accessed on 4 May 2021) to design and synthesize FISH probes [62]. This platform uses OligoArray and UNAfold [63] to design oligos. Oligos that have a single genomic target are identified using BLAST [46]. Oligopaint now hosts oligo-FISH probes for six species (*C. elegans*, *D. melanogaster*, *Arabidopsis*, mouse, zebrafish, and human) [64].

PROBER is specialized for FISH oligo probes design [55]. It first searches for short substrings in a repeat-free genomic region within 10–100 kb based on ‘MerMatch’, an algorithm from ‘MerEngine’ [65]. It then eliminates probes that overlap with repeats in the target regions and calculates the Tm and GC content to avoid non-specific annealing or primer dimerization. The final designed oligo probes range from 100 to 2000 bp and are suitable for oligo-FISH in specific genomic regions. Nevertheless, PROBER is not implemented for genome-wide oligo probe design and the lengths of the designed probes are long, which generate lower and discrete FISH signals in a given chromosome region. This software has been applied for tiling FISH probe design in humans. Some other pipelines using a similar strategy to PROBER have also been used in mammals [66,67,68].

mathFISH [23] and webFISH [56] are two web-based platforms that were developed for oligo-FISH probes design. Both tools are implemented in MATLAB. However, mathFISH is specialized for the evaluation of pre-design oligo probes, which facilitates the selection of the final oligo-FISH probe sets. mathFISH utilizes thermodynamics-based mathematical models to evaluate each input probe sequence along with its target sequences. In addition, the tool provides several sub-modules to analyze free energy, mismatch discrimination and competitors. Unlike mathFISH, webFISH is specialized for genome-wide single-copy and repetitive DNA FISH probes design. It uses Megablast software to align the query sequence to target unmasked genome sequences. Unique sequences are selected for single-copy probes. This tool has been used for FISH studies of interphase nuclei during class switch recombination in human.

With the requirement for high-resolution FISH and the development of synthesis technology, oligonucleotide-based FISH has been greatly improved. The length of oligo probes has become shorter (<100 bp) and the design of oligo probes has entered the high-throughput era. Massive parallel synthesis of probes becomes prevalent. In addition, more and more plant researchers have applied oligo-FISH technology for their cytogenetic studies.

In 2015, the Jiang laboratory developed a bioinformatic pipeline called Chorus, to design and select oligo probes for FISH in plants on a genome-wide scale [8]. The pipeline integrates RepeatMasker (http://www.repeatmasker.org; accessed on 4 May 2021) for repetitive sequences filtering and utilizes BLAT [69] to identify single-copy oligos. Single-copy oligos are further filtered using Primer3 [70] based on the Tm. Probes designed by this pipeline showed high specificity and resolution across the cucumber chromosome 3 [8]. In 2021, the developer of Chorus upgraded their pipeline and named it Chorus2 [51]. Chorus2 has a faster probe design process because of the replacement of BLAT by the NGS aligner BWA [48]. In addition, with the assistance of a k-mer based method and genomic shotgun sequences, probes designed by Chorus2 are more specific and robust than those designed using Chorus. Furthermore, Chorus2 adds several functions to design oligo probes for genetically related species and species without a reference genome. Hoang et al. recently introduced a pipeline that enables oligo probe design for congeneric species [71]. Both methods are suitable for chromosome evolution researches. To date, Chorus/Chorus2 have been applied for various plant cytogenetic studies [8,18,72,73,74,75].

To simplify the design process and improve the specificity of oligonucleotides, Hendling et al. developed a web-based design tool called Oli2go [57]. The web server of Oli2go provides many parameters for users to design probes, check primers and dimers, and enables a specificity check against multiple species based on their genome sequences, whole genome shotgun (WGS) sequences, and environmental samples. Although Oli2go offers an all-in-one solution for probe design, the tool is developed for non-human DNA and is not optimized for FISH oligo probe design.

Beliveau and his colleagues focused on the visualization of chromosome structures and chromatin dynamics. They developed a versatile pipeline, named OligoMiner, for the genome wide design of oligo probes for FISH [21]. OligoMiner provides a streamlined method that integrates probe length, multiple thermodynamics analyses, and specificity for FISH experiments with different requirements. Based on the pipeline, the Wu laboratory re-designed oligo-FISH probes for six species and updated their datasets in Oligopaints [19]. The lack of a graphical user interface (GUI) prompted Passaro et al. to wrap the OligoMiner pipeline into a web-server application called OligoMinerApp [58], which broadens the user community and provides a more convenient experience for oligo-FISH probe design. The OligoMinerApp currently hosts 10 reference genomes for probe design. The Beliveau laboratory also built their own web platform named Paint Server and Homology Optimization Pipeline (PaintSHOP, https://paintshop.io/; accessed on 4 May 2021) for the design of genome-scale oligonucleotide for FISH experiments [60]. This platform updates the backend OligoMiner scripts and provides an all-in-one pipeline from probe design to synthesis.

iFISH is a platform designed specifically for FISH probes selection [22]. It uses pre-designed genome-wide oligo probes from human as a database and selects one or multiple optimal probes in a given region. Oligo probes designed by iFISH showed good results in the visualization of chromosome territories and the quantification of chromosome intermingling in human. To help users better design and select the oligo probes, a freely accessible web interface called iFISH4U (http://ifish4u.org; accessed on 4 May 2021) was also developed.

ProbeDealer is a recently developed tool to design oligo-based FISH probes [59]. It utilizes features such as melting temperature, GC content, secondary structure and dimers to filter oligos detected from genomes based on a sliding window method. A specificity check is performed using BLAST. ProbeDealer is equipped with a user-friendly GUI and is specialized for chromatin tracing and RNA FISH experiments.

Kmasker plants is a tool designed to assess complex plant genome sequences [50]. It also offers a FISH probe analysis function based on a k-mer analysis method to enhance the specificity of the designed oligo probes. This function utilizes WGS data to filter repetitive sequences, a similar method to that performed in Chorus2. Probes selected by Kmasker plants showed robust signals in FISH experiments in *Aegilops speltoides* [76]. The web server of Kmasker plants currently provides 10 plant genomes for FISH analysis.

There are some other platforms that enable oligo probe design, such as commercial probe design platforms from Arbor Biosciences or LGC Biosearch Technologies; however, these tools are not in the scope of this article.

## 4. Evaluation of State-of-Art Tools for Oligo-FISH Probe Design

To fulfill the requirement of different experiments using oligo-FISH, more and more computational tools or platforms for oligonucleotide probe design have appeared and are freely available. Although these tools are implemented to design probes, they show marked differences in their pipelines and functions. In addition, as we discussed above, specificity is the key consideration for a successful oligo-FISH experiment, thus the specificity of the probes designed by these tools needs to be inspected. Furthermore, the practicality and performance of these tools or platforms should also be considered. For example, tools with obscure and awkward pipelines may preclude users from implementing them, especially those users who have little bioinformatic knowledge. Therefore, we chose four commonly used and state-of-art oligo-FISH probe design tools or platforms, and comprehensively discussed and evaluated them. We aimed to provide instructions to scientists who require designed oligo probes for FISH.

### 4.1. OligoMiner

As oligo-FISH became more and more popular for the study of chromosome organization and gene expression, the demand for bioinformatic design utilities for oligo probes increased. Beliveau et al. firstly designed oligo probes using the OligoArray tool [54,61] and built a database called Oligopaints [62] to maintain the designed probes. However, genome-scaled probe design is difficult using OligoArray. To solve this problem, OligoMiner, a rapid and robust computational pipeline, was developed for the genome-scale design of oligo FISH probes [21]. Compared with OligoArray, OligoMiner is superior in terms of speed, number of adjustable parameters, throughput, and probe density. Besides, probes designed by OligoMiner enables highly efficient conventional and super-resolution imaging.

OligoMiner is equipped with python and uses standard bioinformatic file formats at each step in the probe mining process. OligoMiner provides two distinct modes for predicting probe specificity and filtering multi-copy probes. One is named Unique Mode (UM), which aligns all probe sequences to the target genome using bowtie2 and identifies uniquely mapped candidate probes. The other approach is termed Linear Discriminant Analysis Mode (LDA Mode, LDM), which uses supervised machine learning (ML) methods from the scikit-learn package. This mode is implemented to connect probe sequence alignment scores and duplexing probabilities. LDM can identify potentially problematic DNA hybridization effectively as well as the much slower thermodynamic simulations, thus, producing better FISH results.

OligoMiner enables several post-processing functions for designed probes. The kmerFilter function uses Jellyfish [77] to screen probe sequences containing high-abundance k-mers and then filters out these probes because they could lead to off-target binding. For some specific experimental conditions, users can check and filter probes that might form unwanted secondary structures using the structureCheck function.

OligoMiner provides command-line operations that can be performed on Windows, Macintosh, or Linux systems. Additionally, OligoMiner can be directly installed via conda, an open source package and environment management system [78].

### 4.2. iFISH

Unlike OligoMiner, iFISH is an open-source repository that hosts genome-scale oligo-FISH probes for human [22]. In fact, iFISH is an oligo probe selection platform rather than a probe design tool, because it does not start from scratch to design probes. In contrast, iFISH queries a pre-designed oligo probe database, and designs various probes along regions of interested regions in the genome with considerations of target size, homogeneity, centrality and distance between two adjacent probes. However, the authors also introduced a pipeline to build their own oligonucleotide database. We discussed this pipeline below (Section 4.5).

Although iFISH is not implemented for de novo oligo probe design, compared with OligoMiner, iFISH has several advantages. For example, iFISH can control the probe density in a given genomic region of interest, especially for chromosome spotting probes spaced on the same chromosome. This function is very useful for specific FISH experiments. The authors have provided a 40 mer public database, and compared these oligo probes with OligoMiner’s probe datasets. The results showed that the iFISH-40 mer dataset had a higher density (by approximately 2.6 times) than the OligoMiner Balance (OMB) dataset, which indicates that the iFISH dataset is more suited for designing oligo-FISH probes in the human genome, especially for small regions. In addition, the FISH signals produced by iFISH probes were higher than those produced by OMB probes, which proves the importance of probe density in a given region.

Based on selected chromosome spotting probes from iFISH, intermingling of chromosome territories can be easily distinguished. Nevertheless, oligo probes are only designed for human using iFISH, and more probe datasets for other species are needed. In general, iFISH is a valuable platform for oligo-FISH probe design.

### 4.3. Chorus2

Chorus software [8] was initially implemented to design oligo-FISH probe for plants and then updated (named Chorus2 [51]) to design robust oligo probes in plants and other species using k-mer scoring and NGS filtering methods.

Chorus2 and OligoMiner have similar approaches to design genome-wide oligo-FISH probe. They are both built by python and use NGS alignment and k-mer-based methods to filter probes. However, Chorus2 produces more specific probes because it introduces a particular strategy called ChorusNGSfilter. The ChorusNGSfilter function utilizes shotgun sequences data to further filter potential repetitive sequences in pre-designed oligos, using Jellyfish [77] to calculate the k-mer scores of each probe. This is a trade-off between the probe numbers/density and probe specificity; however, it provides highly accurate target signals and low background noises in FISH experiments. Compared with OligoMiner, Chorus2 is designed to generate fewer putative repetitive oligo probes, which demonstrates the robustness of Chorus2.

Except for the genome-wide design of oligonucleotide probes, Chorus2 also allows the design of conserved probes among genetically related species. With the ChorusHomo function, users can find syntenic regions between two species and use the probes designed from these regions for FISH in both genomes. In addition, Chorus2 enables the design of probes for a species without a reference genome. The corresponding function, ChorusNoRef, takes advantage of the reference genome from a closely related species and uses shotgun sequences of the target species to build a pseudo-genome sequence. Chorus2 then designs oligos from the constructed pseudo-genome sequence. With these two functions, oligo-FISH can be applied for further researches.

Chorus2 is available on any modern system and can be installed using conda. Chorus2 also provides a user-friendly GUI for users to design and select probes. Furthermore, the authors offered nine oligo probe datasets for different plant and animal species. With the assistance of the comprehensive documentations and video tutorials, Chorus2 is easily to use for both dry-lab and wet-lab scientists.

### 4.4. PaintSHOP

The Beliveau laboratory recently developed an interactive platform for the reproducible design of oligo-FISH experiments named PaintSHOP, to further improve the design of oligo probes [60]. PaintSHOP can identify probes for different experimental targets efficiently, add necessary sequences such as primer pairs, and finally generate standardized files documenting the design of each library.

PaintSHOP is built with a dynamic web application using the Shiny framework from the R programming language. Similar to iFISH, PaintSHOP is implemented to design probes from pre-built oligo datasets. However, PaintSHOP can also de novo generate genome-wide probes. PaintSHOP inherits the probe mining process of OligoMiner, but it improves some procedures and calculates an on-target score and an off-target score for each probe. PaintSHOP utilizes a machine learning model that was built using the XGBoost library, and uses the length, GC-content, dinucleotide counts, and NGS alignment scores as features to predict probe specificity quantitatively in the whole genome. Moreover, PaintSHOP incorporates genome annotation information to identify probes in genome intervals shared by all transcript isoforms of a given gene. All these processes in the pipeline are integrated into an automated Snakemake workflow [79].

With the improvement of the probe design pipeline, PaintSHOP designed more probes than iFISH4U probes for human, which made up for the deficiency in the OligoMiner-designed probe sets. By contrast, the PaintSHOP resources host FISH probe sets of minor species, and de novo probe design using PaintSHOP requires the knowledge of bioinformatics, such as Snakemake. In summary, PaintSHOP greatly facilitates the usage of oligo probes in transcriptome and genome-scale oligonucleotide FISH experiments.

### 4.5. Different Pipelines of Oligo Probe Design among Different Tools

**Figure 2:**
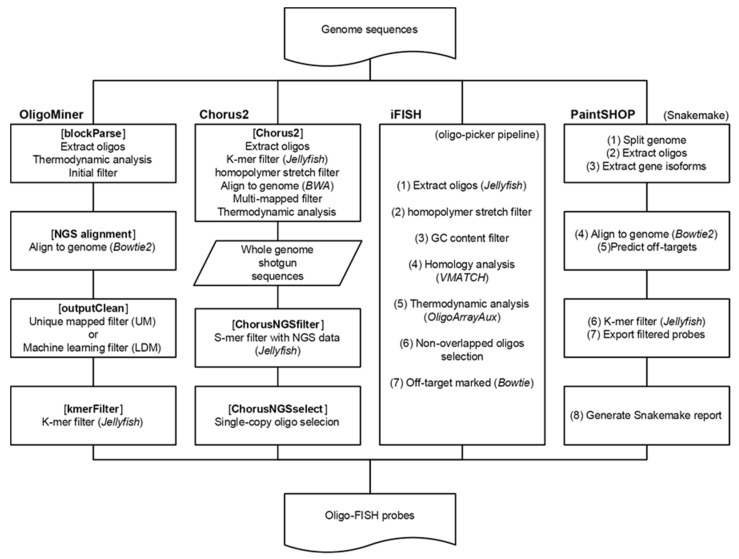
Different pipelines (OligoMiner, Chorus2, iFISH, and PaintSHOP) used for oligonucleotide fluorescence in situ hybridization (Oligo-FISH) probe design.

The pipeline of each tool discussed above are quite different. Thus, we first performed a comprehensive comparison of the design processes among the tools (Figure 2).

OligoMiner requires a single sequence (such as one region in the genome or one chromosome) in the fasta format as the input, and extracts oligo sequences with a given length and thermodynamic properties. After this step, the extracted sequences are aligned to the target genome sequence using NGS aligner Bowtie2 with customized parameters. Output results are then cleaned based on their uniqueness in genome (UM) or on a machine learning model (LDM), which takes the probe length, alignment score, and certain other thermodynamic properties into consideration. Cleaned oligo probes can be further filtered by their k-mer values using Jellyfish to remove sequences with high-abundance k-mers.

iFISH does not provide a pipeline for de novo probe design; however, the authors described the processes by which they built the iFISH-40 mer database. First, unique 40nt sequences from the human genome were extracted using Jellyfish and custom scripts. Then, probes with long homopolymer stretch (≥7) or extreme GC contents (<35% or >80%) were filtered out. Then, probes that had 70% or higher homology to more than one genomic location were detected by VMATCH and discarded. The secondary structure and melting temperature of each probe were calculated. Finally, all non-overlapping probes were aligned to reference genome using Bowtie to check the presence of off-target effects, and only probes with ≤10 off-target sites (mismatches ≤ 5) were retained as clean probes.

Chorus2 follows three major processes to design oligo probes. The first process is to design all oligo probes with a given length from the target genome. Jellyfish is then used to remove putative repeat sequences and filtered oligo sequences from a sliding window are further filtered by the presence of homopolymers and then mapped to target genome by BWA to examine their uniqueness in the genome. The hybrid Tm and hairpin Tm of each oligo are analyzed using Primer3-py. Only probes that mapped to exactly one genome loci and have a *d*Tm (hybrid Tm − hairpin Tm) > 10 °C are kept. The second process utilizes shotgun reads from the target genome to further filter potential multi-copy oligos. In this step, the distribution of the k-mer frequencies from NGS data are counted, and each oligo is assigned a k-mer score that represents their repetitiveness. The last process is to select the non-overlapping single-copy oligos. By default, oligos with a k-mer score between the 10% and 90% quantiles are retained. These selected oligo probes can be finally synthesized and maintained as permanent oligo resources.

PaintSHOP takes advantages of the OligoMiner pipeline with slight modifications. It identifies all possible probes between 20 and 60 nucleotides in length with a Tm between 42 and 47 °C in soft-masked genome regions by default. A new automatic model selection and hyperparameter optimization is performed using TPOT, a method converged on a XGBoost regressor, to simulate the potential “probe-target” relationship. PaintSHOP calculates the on-target and off-target score of each oligo based on machine learning results. Finally, probes are filtered by their k-mer scores and annotated with reference annotation information.

### 4.6. Performance of Probe Design Pipelines among Different Tools

To obtain a more comprehensive understanding of each tool, we performed oligo-FISH probe design using these tools on a local server (see Supplementary Materials), and then we evaluated the performances of these tools and the properties of the probes generated by them.

**Figure 3:**
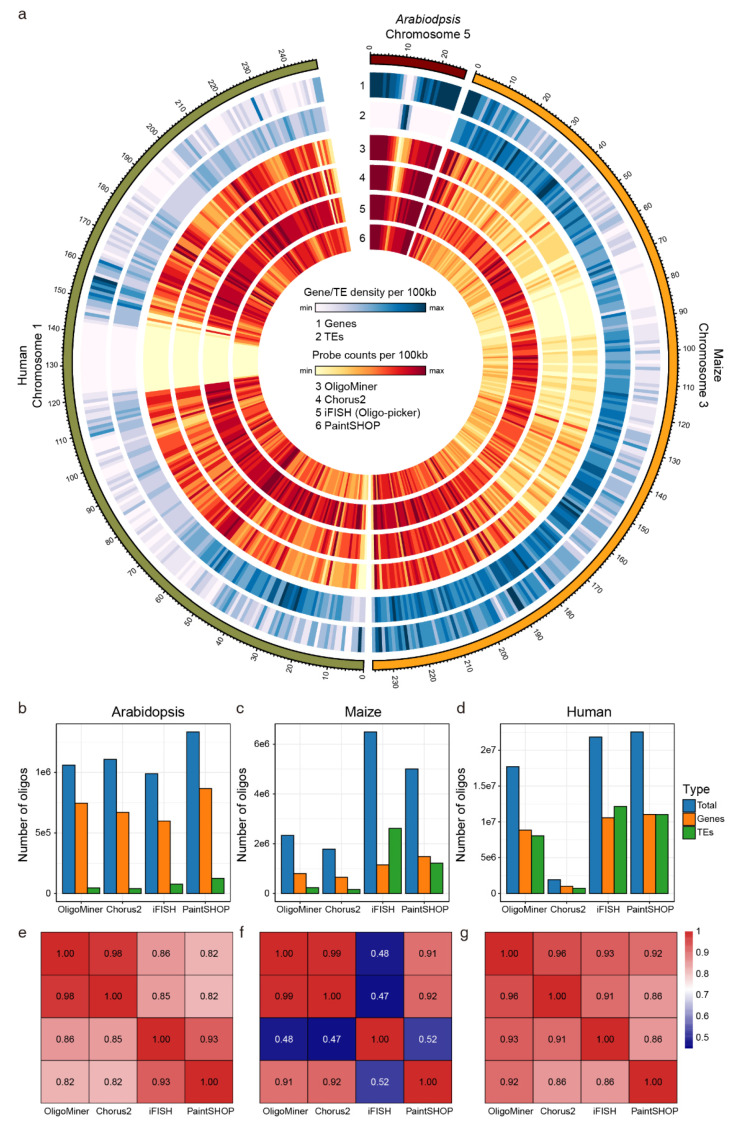
Comparison of probes designed using state-of-art tools. (**a**) Distribution of probes designed using the four tools in *Arabidopsis* chromosome 5, maize chromosome 3 and human chromosome 1. (**b**–**d**) Number of oligo probes designed for *Arabidopsis* (**b**), maize (**c**), and human (**d**) located in gene body or transposable element (TE) regions. (**e**–**g**) Pearson correlation of the density of oligo probes for *Arabidopsis* (**e**), maize (**f**), and human (**g**).

We used all four tools to design oligo-FISH probes for three species (*Arabidopsis*, maize and human). The tools were run using their default parameters or instructive parameters. The length of the designed oligo probes was set as 45nt for all tools. We first compared the number of oligo probes designed by each tool in the three species. In *Arabidopsis* genome, the four tools developed a similar number of probes, which may be due to the small size of the genome (Table 2, Figure 3b–d). However, the number of probes designed in the maize and human genomes varied markedly among the different tools. In the maize genome, OligoMiner and Chorus2 generated fewer oligo probes than iFISH and PaintSHOP. In the human genome, the number of probes designed by Chorus2 was one order of magnitude less than that designed by the other tools, and PaintSHOP generated the largest number of probes. The results indicated that Chorus2-designed probes might be not a good choice for targeting small regions in the human genome.

Next, we compared the density of probes designed by the four tools in different genomes. We first counted the coverage of probes designed by the four tools in several genomic windows (Table 3). Probes designed in the *Arabidopsis* genome showed high and similar densities in all the four tools. However, in maize, the coverages of probes in all these tools were sparse, which were affected by the highly repetitive genome. Chorus2-designed probes had relatively lower coverage in the human genome, which was consistent with the total number of probes designed by Chorus2. Overall, iFISH or PaintSHOP designed probes had the highest coverage among all the three genomes. We next plotted a genome-wide distribution of genes, transposable elements (TEs) and oligo probes in the three chromosomes from different genomes (Figure 3a). The plot showed that probes designed by all the tools had similar distributions. The densities of the probes were relatively lower in the TE enriched regions, proving that these tools can remove repetitive sequences in the probe sets (Figure 3b–d). We calculated the Pearson correlations of probe density (probes per 100 kb non-overlapping window) among the four tools (Figure 3e–g). This analysis showed that OligoMiner and Chorus2-designed probes had similar distributions in all three genomes, while iFISH did not correlate well with the other tools in maize. Besides, more iFISH designed probes covered TE regions than gene enriched regions (Figure 3c). The results indicated that the iFISH pipeline may be not efficient to eliminate repeats in genomes with abundant repetitive sequences.

We also compared the speed and memory usage of these tools (Table 2). The pipeline introduced by iFISH was originally performed using high performance computing (HPC), thus we ran the pipeline manually step-by-step on our local server. Among all the tools, Chorus2 ran fast for all species, followed by PaintSHOP. The iFISH pipeline ran over 1 day for maize and human. For memory occupancy, iFISH and PaintSHOP used the most, followed by Chorus2 and OligoMiner. PaintSHOP used over the maximum memory of our test server. In general, OligoMiner and Chorus2 software are suitable for oligo probe design on a personal computer, while iFISH and the PaintSHOP pipeline might require one or more high performance server(s).

## 5. Discussion and Conclusions

Oligonucleotide-based FISH technology has been applied for various research areas, including traditional cytogenetic studies and burgeoning 3D genome researches [7,8,18,19,22,64,72,73,80]. With the help of advanced technology, the mysteries of genomes are gradually being brought to light. However, all these studies require robust oligo probes to identify the specific genomic regions. Many oligo probe design tools and platforms have been developed to cope with fast-growing demands. In the present review we highlighted the key aspects that should be considered during the probe design process and reviewed the development of current tools for oligo-FISH probe design systematically. Each tool utilizes its own principle to design and filter oligos in target genomes. The selection of an optimal tool for FISH probes design is important. For instance, detection of chromosome variation and evolution requires a large number of probes to cover the target chromosome, thus tools used for probe design in small regions are not suitable for this job. Thermodynamic parameters are of concern for some FISH experiments that use different conditions in the experiments, such as temperature and salt concentrations. The stability of hybridization between the probe and its target might be impaired under different conditions [30]. For FISH experiments in plant species, whose genomes comprise large amounts of repetitive sequences, improving the specificity of oligos in the genome is the principal consideration. Traditional strategies based on alignment may not eliminate repeats well [51]. Combination of alignment-based and k-mer-based methods shows improved specificity of oligos in genome. Nevertheless, this method may not work well in repeat-rich genomes, which usually have low quality reference sequences because of the difficulty of genome assembly. Genomic shotgun sequences are great resources for repeats filtering during probe design. De novo identification of repetitive sequences using RepeatExplorer and k-mer-based methods with the assistant of WGS data both exhibited high efficiency in removing potential repeats [50,51,71].

We reviewed and compared the four latest developed tools that enable genome-scaled oligo probe design comprehensively. From their documentation and our evaluation results, we offer some instructions for choosing which tool to use: OligoMiner and Chorus2 software are suitable for users who do not have high performance computers. OligoMiner is preferred for mammals and Chorus2 is preferred for plants. For wet-lab scientists, the web server OligoMinerApp is a good choice to develop probes in the 10 provided genomes. The authors of Chorus2 also provide well-rounded documentations and video tutorials to follow. iFISH is used for chromosome spotting or obtaining uniform probes in regions of interest in human. PaintSHOP is recommended for users who want to design and select oligo probes without complicated considerations. Users can select pre-designed probes from in the probe sets hosted by PaintSHOP.

The present review may not cover all the tools used for oligo-FISH probe design, and many oligo design tools were not evaluated; however, we have summarized the generalized principles to design oligo probes and marked the features of each tool in Table 1. We hope that this review provides useful suggestions for scientists when choosing a suitable tool. We also anticipate that our review will broaden the application of oligo-FISH for more studies, and provide some guidance for probe design and the development of related software.

## Figures and Tables

**Table 1:** General information of several oligo probe design tools.

Tools	Year	Platform	Language	Aligner	Features
OligoArray [54]	2003	Linux	Java	BLAST	Tm, secondary structure, specificity
PROBER [55]	2006	Windows, Web	C#	MerEngine	Tm, specificity, tiling oligo probes
mathFISH [23]	2011	Web	MATLAB	ClustalW	Thermodynamics, mismatch
webFISH [56]	2012	Web	MATLAB	Megablast	Specificity, homology, user-friendly
Chorus [8]	2015	Linux, MacOS	Python	BLAT	Genome-scale, specificity, homology, plants
Oli2go [57]	2018	Web	-	BLAST, BWA	Specificity, thermodynamics, user-friendly, non-human
OligoMiner [21]	2018	Linux, Windows, MacOS	Python	Bowtie2	Genome-scale, specificity, thermodynamics, machine-learning, fast
iFISH [22]	2019	Linux, Web	Python, Perl	Vmatch, Bowtie	Genome-scale, pre-designed, selection, user-friendly, human
Kmasker plants [50]	2020	Linux, MacOS, Web	Perl, R, Python	BLAST	Specificity (WGS), plants
OligoMinerApp [58]	2020	Web	Python	Bowtie2	Genome-scale, specificity, thermodynamics, machine-learning, user-friendly
ProbeDealer [59]	2020	Windows, MacOS	MATLAB	BLAST	Genome-scale, specificity, thermodynamics, user-friendly
Chorus2 [51]	2021	Linux, Windows, MacOS	Python	BWA	Genome-scale, specificity (WGS), homology, fast, comparative analysis, plants
PaintSHOP [60]	2021	Linux, MacOS, Web	Python, R	Bowtie2	Genome-scale, specificity, thermodynamics, machine-learning, user-friendly

**Table 2:** Performance comparison of four tools and the probes designed by the tools.

		OligoMiner	Chorus2	iFISH	PaintSHOP
**Number of oligo probes**	*Arabidopsis*	1,059,677	1,107,815	989,167	1,333,798
	Maize	2,339,006	1,780,857	6,489,281	5,003,474
	Human	17,717,778	1,940,978	21,847,197	22,555,306
**Running time (min)**	*Arabidopsis*	43.5	44.2	-	25.9
	Maize	1038	381.7	-	Failed on local server
	Human	1311	243.3	-	
**Max memory usage (GB)**	*Arabidopsis*	3.4	9.1	-	12.1
	Maize	28.5	24.4	-	Over 64 GB
	Human	25.2	34.8	-	
**Adjustable probe length**		√	√	√	√
**Thermodynamics analysis**		√	√	√	√
**GC content selection**		√	×	√	√
**Control distance between probes**		√	√	√	×
**De novo** **probe design**		√	√	Upon request	PaintSHOP_Snakemake
**Further probe selection**		×	ChorusPBGUI	iFISH probe design	PaintSHOP
**Have GUI**		OligoMinerApp	ChorusGUI	iFISH4U	PaintSHOP
**Characteristic**		Machine-learning for temperature-specific probe design	Specialized for plants, probe design for closed related species and species without reference genome	Probe selection based on target size, centrality and homogeneity	One-step design and selection of oligo probes, enable appending primers and bridge sequences

**Table 3:** Coverage of probes designed by the four tools in genomic windows.

Species	Genome Window	OligoMiner	Chorus2	iFISH	PaintSHOP
*Arabidopsis*	1:4,000,000-6,000,000	10.706/kb	10.923/kb	9.056/kb	12.205/kb
	5:20,000,000-22,000,000	10.014/kb	10.640/kb	8.389/kb	11.375/kb
Maize	1:230,000,000-235,000,000	1.666/kb	1.293/kb	3.378/kb	3.082/kb
	3:185,000,000-190,000,000	1.675/kb	1.288/kb	3.280/kb	3.125/kb
Human	chr1:40,000,000-45,000,000	6.550/kb	0.743/kb	8.228/kb	7.736/kb
	chr19:10,000,000-15,000,000	3.652/kb	0.519/kb	6.845/kb	4.874/kb

## Data Availability

The data analyzed in this study are openly available and stated in Supplementary Materials.

## Supplementary Materials

The following are available online at https://www.mdpi.com/article/10.3390/ijms22137124/s1; contains references [81,82,83].
