# Supplementary material for: Single Copy Oligonucleotide Fluorescence In Situ Hybridization Probe Design Platforms: Development, Application and Evaluation
Source: Int J Mol Sci. 2021 Jul 1;22(13):7124. doi: 10.3390/ijms22137124 (PMC8268824; doi:10.3390/ijms22137124)
Supplement: Supplementary file 1 [file ijms-22-07124-s001.zip › ijms-1261851-supplementary.pdf]

Supplementary Methods

For comparison among four oligo-FISH probe design tools [1-4], we ran the pipelines on a local server, which has 16 CPUs and 64GB of memory. The system of the server is CentOS 7.9. iFISH and PaintSHOP were rerun on a CentOS server with 48 CPUs and 512GB of memory because of memory overflow. All the tools and their dependent environments were installed via bioconda [5]. OligoMiner was run according to instructions from https://github.com/beliveau-lab/OligoMiner (accessed on 4 May 2021). Chorus2 was performed with default parameters. The iFISH probe design pipeline was run with the adapted scripts from oligo-picker (https://github.com/ggirelli/oligo-picker; accessed on 4 May 2021). PaintSHOP was performed using the Snakemake [6] pipeline (https://github.com/beliveau-lab/PaintSHOP_pipeline; accessed on 4 May 2021). The oligo length parameter was set to 45 in all tools. The shotgun reads used by Chorus2 were downloaded from the National Center for Biotechnology Information database Sequence Read Archive under accession number SRR6750188 (*Arabidopsis*) [7], ERR3288216 (maize) [8], and SRR1295425 (human) [9].

References

1. Beliveau, B.J.; Kishi, J.Y.; Nir, G.; Sasaki, H.M.; Saka, S.K.; Nguyen, S.C.; Wu, C.T.; Yin, P. OligoMiner provides a rapid, flexible environment for the design of genome-scale oligonucleotide in situ hybridization probes. *Proc. Natl. Acad. Sci. USA* **2018**, *115*, E2183–E2192, doi:10.1073/pnas.1714530115.
2. Gelali, E.; Girelli, G.; Matsumoto, M.; Wernersson, E.; Custodio, J.; Mota, A.; Schweitzer, M.; Ferenc, K.; Li, X.; Mirzazadeh, R.; et al. iFISH is a publically available resource enabling versatile DNA FISH to study genome architecture. *Nat. Commun.* **2019**, *10*, 1636, doi:10.1038/s41467-019-09616-w.
3. Hershberg, E.A.; Close, J.L.; Camplisson, C.K.; Attar, S.; Chern, R.; Liu, Y.; Akilesh, S.; Nicovich, P.R.; Beliveau, B.J. PaintSHOP enables the interactive design of transcriptomeand genome-scale oligonucleotide FISH experiments. *bioRxiv* **2021**, doi:10.1101/2020.07.05.188797.
4. Zhang, T.; Liu, G.; Zhao, H.; Braz, G.T.; Jiang, J. Chorus2: Design of genome-scale oligonucleotide-based probes for fluorescence in situ hybridization. *Plant Biotechnol. J.* **2021**, doi:10.1111/pbi.13610.
5. Grüning, B.; Dale, R.; Sjödin, A.; Chapman, B.A.; Rowe, J.; Tomkins-Tinch, C.H.; Valieris, R.; Köster, J.; The Bioconda Team. Bioconda: Sustainable and comprehensive software distribution for the life sciences. *Nat. Methods* **2018**, *15*, 475–476, doi:10.1038/s41592-018-0046-7.
6. Koster, J.; Rahmann, S. Snakemake-a scalable bioinformatics workflow engine. *Bioinformatics* **2018**, *34*, 3600.
7. Weng, M.L., Becker, C., Hildebrandt, J., Neumann, M., Rutter, M.T., Shaw, R.G., Weigel, D; Fenster, C.B. Fine-grained analysis of spontaneous mutation spectrum and frequency in *Arabidopsis thaliana*. *Genetics*, **2019** 211, 703–714.
8. Jiao, Y.; Peluso, P.; Shi, J.; Liang, T.; Stitzer, M. C.; Wang, B.; Campbell, M. S.; Stein, J. C.; Wei, X.; Chin, C. S.; et al. Improved maize reference genome with single-molecule technologies. *Nature* **2017,** *546*, 524–527.
9. Genomes Project, C.; Auton, A.; Brooks, L. D.; Durbin, R. M.; Garrison, E. P.; Kang, H. M.; Korbel, J. O.; Marchini, J. L.; McCarthy, S.; McVean, G. A.; et al. A global reference for human genetic variation. *Nature* **2015**, 68–74.
